# *Massilia timonae* bacteremia: An unusual pathogen of septic abortion

**DOI:** 10.1016/j.idcr.2022.e01592

**Published:** 2022-08-02

**Authors:** Gawahir A. Ali, Emad B. Ibrahim, Sanjay H. Doiphode, Wael Goravey

**Affiliations:** a Department of Infectious Diseases, Communicable Diseases Centre, Hamad Medical Corporation, Doha, Qatar; b Department of Laboratory Medicine and Pathology, HMC, Doha, Qatar

**Keywords:** *Massilia timonae*, Bacteremia, Septic abortion

## Abstract

*Massilia timonae* infections in humans have rarely been reported. To the best of our knowledge, *M. timonae* has not been previously recognized as a causative agent of obstetric or gynecological infections. Timely identification of this unusual pathogen and the use of targeted antimicrobial therapy are crucial to avoid consequences and treatment failure.

A 29 year old woman presented with fever and lower abdominal pain for three days. She had an uneventful emergency evacuation of retained products of conception following an incomplete miscarriage and bleeding 10 days before this presentation. Examination revealed pyrexia of 38.4 °C, mild tenderness but soft lower abdomen and noticeable cervical motion tenderness. Investigations showed WBCs of 20.3 × 10^9^/L (4–11.0), CRP 251 (0–5 mg/L), Procalcitonin 6 µg/L (< 0.05), and 2.4 × 1.3 cm remnant or blood clots were seen ultrasonography within the uterine cavity (Fig. 1). Therefore, piperacillin/tazobactam was started empirically and 2 days later, non-fermentative gram-negative bacilli grew from four bottles of blood culture and a high vaginal swab (Fig. 2A and 2B). Subsequently, MALDI (TOF-MS, Billerica, MA, USA) identified the organism as *Massilia timonae.* The fever resolved quickly, and the antimicrobial was switched to ciprofloxacin according to sensitivity (Table 1). Follow-up US of the pelvis showed no evidence of previously reported remnants; thus, ciprofloxacin was continued for 14 days after confirming the negativity of the blood cultures. At two weeks of clinical follow-up, she was well and reported no recurrence of fever.

**Fig. 1:**
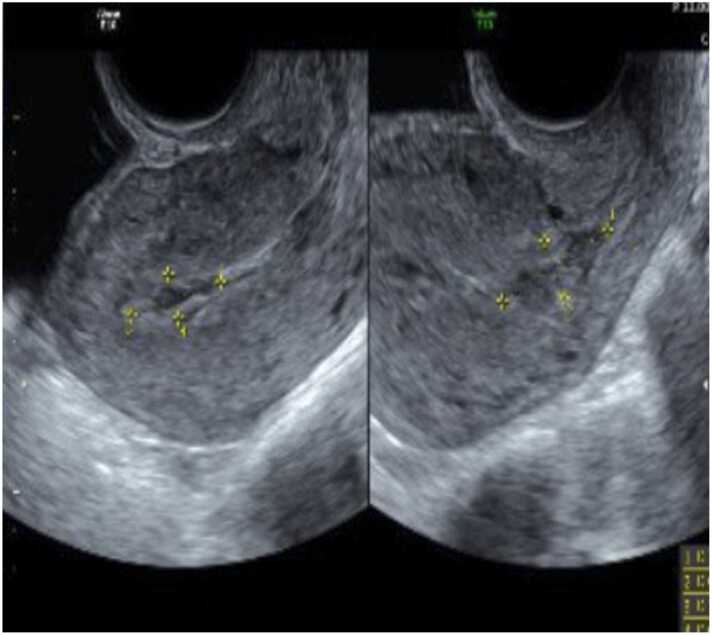
US pelvis showing 2.4 × 1.3 cm remnant/blood clots within the uterine cavity.

**Fig. 2:**
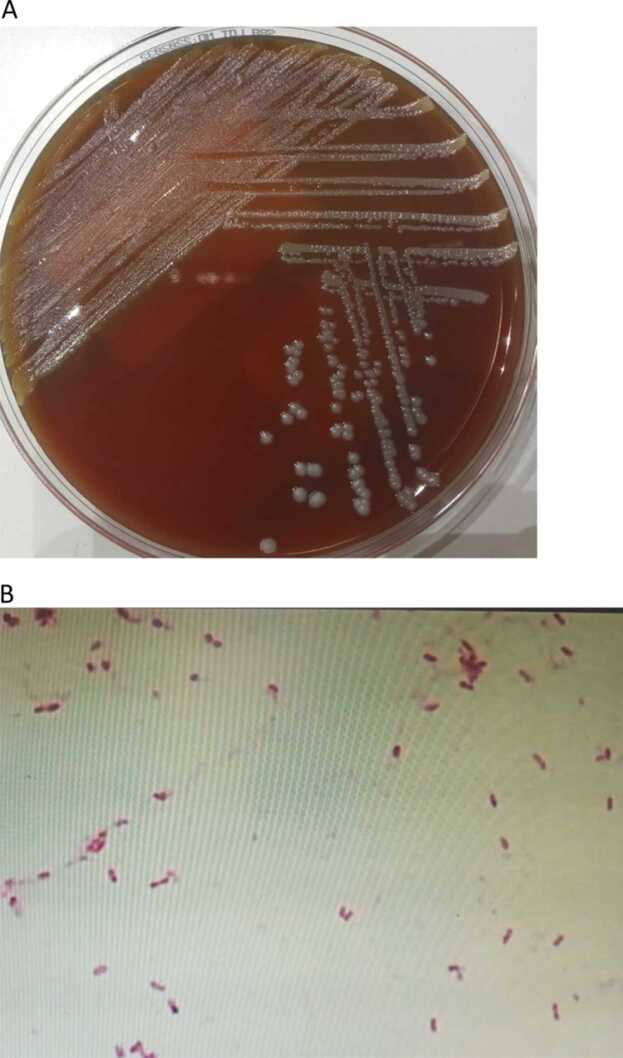
(A) Grayish, glistening mucoid colonies of *M. timonae* on blood agar. (B) Gram stain revealed abundant gram-negative straight rods.

**Table 1:** Antimicrobial susceptibility of the isolated Massilia timonae (BD Phoenix, BD Biosciences, Heidelberg, Germany) according to CLSI.

Antibiotics	Interpretation
Ciprofloxacin	S
Ceftriaxone	R
Gentamycin	S
Amikacin	I
TMP/SMX	R
Piperacillin/Tazobactam	S
Meropenem	S

S = Susceptible; I = Intermediate; R = Resistant.

*Massilia timonae*, an aerobic gram-negative bacillus belonging to the family *Oxalobacteraceae*, was first described in 1998 and since then only a few cases have been reported [1]. Therefore, the clinical characteristics and outcomes of this pathogen remain unclear due to the limited knowledge. The clinical syndromes reported to date include bacteremia, CNS infections, wound infections, lymphadenitis, and osteomyelitis, and the infection is not limited to an immunocompromised status [2]. However, no studies have identified *M. timonae*-related septic abortion, which is typically caused by polymicrobial bacteria and originates from the genitourinary flora [3]. Interestingly, no confirmed source of the infection has been identified for all cases so far, but few have suggested *M. timonae* may be an environmental organism or occur as part of the transient normal flora [1], [4]. The latter explanation may be plausible for our patient. Importantly, treatment should be guided by the pattern of antimicrobial sensitivity and the optimal treatment duration remains unknown [1]. Early diagnosis and effective therapy of *M. timonae*-related septic abortion are essential for improving prognosis and avoiding devastating consequences [3], [4].

## CRediT authorship contribution statement

**GA:** Clinical management, data acquisition and manuscript writing. **EI** and **SD** Microbiological reports. **WG:** Corresponding author, Clinical management, contribute to data acquisition, manuscript preparation and final proof reading.

## Ethical approval

Ethics approval and permission was obtained to publish the case reports from the institutional review board which is in line with international standards.

## Consent

A written informed consent was obtained from the patient to include clinical presentation together with results and imaging. This was subsequently reviewed and approved by the institution ethics and research review board with MRC-04-22-425.

## Funding

No funding was received towards the publication.

## Conflict of interest

The authors declare that they have no competing interests.

## Data Availability

The authors confirm that the datasets supporting the findings of this case are available from the corresponding author upon request.
